# Management of Eustachian Tube Dysfunction: A Review

**DOI:** 10.7759/cureus.31432

**Published:** 2022-11-13

**Authors:** Rajeshwaree Bal, Prasad Deshmukh

**Affiliations:** 1 College of Medicine, Jawaharlal Nehru Medical College, Datta Meghe Institute of Medical Sciences, Wardha, IND; 2 Head and Neck Surgery, Jawaharlal Nehru Medical College, Datta Meghe Institute of Medical Sciences, Wardha, IND

**Keywords:** eustachian tube anatomy, eustachian tube dysfunction questionnaire, eustachian tube pathology, eustachian tube function, eustachian tube dysfunction improvement

## Abstract

The Eustachian tube is a crucial pneumatic component of the head and neck region and is often neglected as an important site of insidious pathologies. In our literature review, we negotiate the management of eustachian tube dysfunction and explore the various therapeutic and surgical options available at our disposal. We begin by investigating the physiological nature of the eustachian tube and its role in the body's functioning. We also list and elaborate on the various pathologies affecting the Eustachian tube and its associated structures. The review then outlines eustachian tube dysfunction and discusses the pathophysiology involved in the genesis of the condition and its progression. Further, the review explores the tools most commonly used to diagnose or alleviate the condition, including, but not limited to, the Valsalva maneuver, Toynbee maneuver, tympanometry, pressure chamber test, and video nasopharyngoscopy. We also touch on The ETS-7 questionnaire and then on the various surgical interventions that may be used to manipulate the condition. The review also describes conventional tympanostomy and myringotomy, along with more novel techniques such as microwave ablation, laser eustachian tuboplasty, and balloon eustachian tuboplasty. We conclude by establishing the most favorable course of treatment in cases of eustachian tube dysfunction.

## Introduction and background

The middle ear is connected to the nasopharynx by the bone-muscle pipeline known as the eustachian tube, which regulates intermediate ear pressure, clears foreign objects, and protects the eardrum [1]. Dysfunction in the eustachian tube forms the pathological foundation of various middle ear diseases like cholesteatoma, chronic otitis media with effusion, tympanic membrane perforations, and cartilage malfunction.

The eustachian tube has two primary functions: balancing the pressure between the middle ear and the surroundings and helping facilitate the secretions from the middle ear, as well as protecting the ear from loud and potential sound hazards. Muscles responsible for these functions are tensor veli palatini and levator veli palatini. Although they don't function similarly, their simultaneous contraction opens the tube. As the tube empties, pressure equalization between the middle ear and the atmosphere takes place. Moreover, facial movements like deglutition and yawning cause muscle contraction that is connected to the eustachian tube, allowing a small amount of air into the tube [2]. However, other functions like aiding in draining the middle ear secretions and their clearance from the ear cavity [3].

Anatomically, the eustachian tube is a hollow tube in bone and acts as a void in fibroelastic cartilage. It is in proximity to the infratemporal fossa and is situated in the para-pharyngeal region. Along the medial pterygoid plate's posterior edge, the eustachian tube runs from the middle ear's front wall to the sidewall of the nasopharynx [4].

Physiological and anatomical defects during intra-uterine life may play a role in eustachian tube dysfunction, due to which difficulty in managing and maintaining patency is observed by surgeons. Some variants from normal anatomy include reduced tube diameter, elevated mucus production, and the tubes becoming blocked due to hypertrophy of the mucosal lining of the tube and slacking of the cartilage around the tube or the tissues [4]. Functional defects may also be brought on by a physical obstruction in the tube, such as a big adenoid pad in the nasopharynx or a blocked or stenotic eustachian tube [5]. Patulous eustachian tube obstruction due to congenital anomalies like cleft palate also affects tube functions. Desquamating epithelium may result in keratoma, causing cholesteatoma of the middle ear blocking the eustachian tube [6].

Eustachian tube or auditory tube dysfunction implies improper functioning of eustachian tube roles, primarily drainage of middle ear secretions, equalizing pressure difference between environment and body, and protection from potentially deafening sounds. The patient must display signs of pressure imbalance in the affected ear, precisely signs of fullness in the ear, popping, and discomfort/pain. Patients may experience additional symptoms, including pressure, a congested or "underwater" sensation, crackling and/or buzzing in ears, auto-phony, and obscured/muffled hearing [7]. When the eustachian tube is dysfunctional, the middle ear develops negative pressures that cause fluid to transude an inflammatory reaction [8].

In the pediatric population, causes of the eustachian tube vary as compared to adults, but the presentation is more or less the same. Several distinctions exist between the ET in children and adults alike when compared to adults, a child's tube has a lengthier osseous component than an adult's. The ET is more horizontal in youngsters. In a newborn, the tube is inclined by about 10° with respect to the horizontal level. The osseous-cartilaginous junction also occurs inline in children. Due to these variations, the tensor veli palatini muscle's attachment to the cartilage of the tube is altered, which reduces the effectiveness of the tube's active opening in young kids. Children are far more likely to contract viral infections, which can hinder both morphological and functional growth. Mucus production, mucosal edema, submucosal lymphoid tissue hyperplasia, and temporary ciliated epithelium injury all contribute to this. Additionally, gastric reflux and allergic rhinitis can cause ET dysfunction. In infants, negative pressure in the middle ear can result from bottle feeding and pacifier use, especially if breathing via the nose is restricted. Children present with chronic otitis media with/without effusion and may have conductive hearing loss [9].

Although, in a definite sense, pressure dysregulation is diagnosed more often as ET dysfunction due to its dominant function in roles of ET dysfunction can be categorized into three types: obstructive, baro-challenged-induced, or patulous dysfunction.

Baro-challenged-induced dysfunction symptoms are well seen when there is an ambient pressure change in the environment, with no otherwise complaints. Patulous dysfunction has similar complaints but with one classic sign of autophony. Obstructive or acute dilatory dysfunction follows after allergic sinusitis/rhinitis or upper respiratory tract infections [10]. Long-term infection of the middle ear (COM) with tympanic damage, inflammation of the middle ear with discharge (OME), and prolonged atelectasis of the ear canals all seem likely to be the result of prolonged disorders of the eustachian tube dysfunction [11].

## Review

Management of pharyngotympanic tube dysfunction

Diagnosis of obstructive dysfunction of the pharyngotympanic tube is currently not supported by a single, gold-standard test, making it difficult for doctors to screen patients for the condition and monitor patient response to treatment [12]. Diagnosis of the type of ET dysfunction depends on history and physical examination. Otoscopy and tympanometry are used to assess pressure changes clinically [10]. This indicates the capability to inflate the middle ear automatically during a Valsalva or Toynbee maneuver to a certain level of the patent eustachian tube. However, this test's sensitivity and specificity basis are still under scrutiny. For assessing pharyngotympanic tube function, the eustachian tube scoring questionnaire (ETS-7) and computerized tomography (CT) paired with the Valsalva maneuver has been suggested as the two primary diagnostic methods [13]. However, the ETS-7 questionnaire is based on patient complaints and historical accuracy rather than a mechanical diagnostic tool which makes it subjective.

Valsalva and Toynbee Maneuver

When doing the Valsalva maneuver, the air is directed to ETS and is intended to aid their opening by sealing the mouth and nose while blowing. The tympanic membrane should move while the patient swallows with their nostrils closed for the Toynbee maneuver to be positive [14]. A positive Valsalva and Toynbee test indicates a partially functioning eustachian tube but is a non-dependable confirmatory diagnostic test due to the precision and expertise required for a correct technique of the maneuvers.

Tympanometry

It is used for the evaluation of pressure and mobility presence of fluid in the middle ear. In normal conditions, pressure should be around zero.

ETS-7 Questionnaire

**Figure 1:**
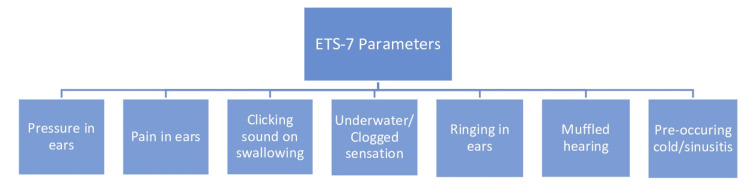
Parameters of ETS-7 Questionnaire of Eustachian Tube Dysfunction

Seven parameters are included for calculating the ETS-7 score: clicking sound when swallowing, pain and pressure in ears, underwater or clogged sensation, pre-occurring cold/sinusitis, ringing in ears, and muffled hearing. Figure 1 depicts all these parameters used for evaluating the score.

Subjective analysis from Toynbee's maneuver and objective Valsalva, tympanometry, and tubomanometric measurements are also used for investigations [13]. A minimum score is considered seven, and a maximum is 49. It was deemed typical to have a total score of less than 14.5 or a mean score of less than 2.1 [15].

Pressure Chamber Test

The subject is instructed to swallow at various pressure controls while in a hyperbaric room, and tympanometry readings of middle ear pressure are recorded. A medial otic force indicates an aberrant tubal function beyond the range of +50 to 100mm pressure exerted by water [16].

Video Nasopharyngoscopy

This technique, which is an adjunct to tympanometry, involves meticulously analyzing the process of dilatation and researching physiology and pathology while performing a trans-nasal endoscopic examination of the nasopharyngeal opening of the eustachian tube while at rest and while swallowing [16].

Non-Surgical Interventions

The evidence from non-surgical studies is generally weak because of the small number and size of the studies, as well as several significant limitations, including inadequate reporting of study design elements, patient characteristics and outcomes data, and short follow-up periods. However, several pharmacological treatments, which include nasal steroids and manual pressure equalization devices, have been proposed and used experimentally [17].

Surgical Interventions

Modern techniques, including laser eustachian tuboplasty and balloon eustachian tuboplasty (BET), have been introduced in addition to conservative methods like tube catheterization and bougie insertion for chronic obstructive tube dysfunction [18].

Conventional Tympanostomy and Myringotomy

Myringotomy and tympanostomy tube insertion in the tympanic membrane are conventional surgical procedures for treating ET dysfunction (TM). With this method, the ET is effectively avoided while middle ear pressure can be equalized, and fluid can be drained via the TM. This strategy successfully treats symptoms but does not address the functioning of the ET. If ET dysfunction continues, tympanostomy tubes frequently need to be replaced more than once. This strains the healthcare system and increases patient dissatisfaction and discomfort. Tympanostomy tubes also carry a small risk of conductive hearing loss due to tympanic membrane perforations. There are now other cutting-edge surgical treatments that concentrate on the ET itself [19].

Microwave Ablation (MWA)

The ET orifice's hypertrophic tissue is the focus of MWA tuboplasty. Overgrown mucosa and submucosa destruction begin via the primary margin of the middle cartilaginous lamina along the unattached margin of the posterior cushion of the pharyngotympanic tube. A straight suction or microwave antenna tip could be used to palpate the ET cartilage easily. On the rear cushion, several pointed tissue ablations must be carried out. In reports of intensely overgrown mucosa, the zone of destruction extends from the ET eustachian tube orifice into the posterior wall of the cartilaginous tubal lumen. Depending on the size of the treated region, several ablation applications are to be done until the ablated zone appears gray-white [20].

Laser Eustachian Tuboplasty

A combined endoscopic nasal and transoral technique performs unilateral or bilateral laser eustachian tuboplasties on the eustachian tube nasopharyngeal opening while under general anesthesia. The tube is filled and fully dilated by employing carbon dioxide or a 980-nm diode laser to vaporize the mucosa and cartilage from the posterior luminal wall [21]. In certain patients with chronic persistent eustachian tube dysfunction, LETP, in conjunction with adequate medical care, maybe a helpful treatment [22]. Under local anesthesia, a marginal level of invasive contouring of the hyperplastic nasopharyngeal Eustachian tube appears to help resolve complaints and eustachian canal function that go along with it, such as improper pressure equalization, auditory fullness, and conductive hearing loss [23].

Balloon Eustachian Tuboplasty

After the standard otorhinolaryngological examination of the nose and rhino pharynx, a trans-nasal endoscopic assessment and microscopic inspection of the ears before the surgery. All patients undergo the Valsalva maneuver. Additionally, audiometric tests should be performed on all patients [24].

A balloon catheter should be introduced into the eustachian tube using a particular insertion device during balloon dilatation tuboplasty under the supervision of a rigid nasal endoscope. The catheter is supposed to be inserted through the ET's nasopharyngeal opening subsequently. The balloon is then inflated with sterile water at the desired pressure for a predetermined time using a pressure device or inflation pump [25]. Stenting materials used are also another area of research. Some that can be tested are polyethylene, silastic, and polyurethane [26]. The most significant decrease in middle ear pressure was observed between catheter insertion and inflation, while the most significant increase was observed between post-inflation and over a minute of inflation [27].

We can conduct balloon dilation tuboplasty in two ways: Trans-tympanic route and trans nasally. The eustachian tube can be balloon-dilated either trans-nasally, the more popular and well-known, or trans-tympanic course, which has less reliable data to back it up to this point. Nevertheless, the risk of carotid artery injury is of utmost significance. In the trans-tympanic route method, the carotid artery damage, by virtue of its anatomy, is a high risk consequence, as is the requirement to elicit a tympano-meatal folding for easy manipulation of the pro-tympanum [28]. Regardless, it has been demonstrated that balloon catheter dilatation of the ET's nasopharyngeal orifice is practical and convenient [29].

Histopathological changes observed after balloon dilatation tuboplasty are seen as the main reduction in inflammatory epithelium alterations and submucosal inflammatory infiltration. The basal layer is typically spared by the balloon, allowing for quick healing. However, the parts of the inflammatory epithelial tissues may be damaged or punctured by the balloon. It also seems to effectively crush lymphocytes and lymphocytic follicles, which could lead to a thinner fibrous scar replacing them. The ET's histopathology after balloon dilation showed changes that might dramatically reduce the impact of inflammation and may help enhance ET function clinically [30]. 

Minor complications after tuboplasty can be surgical emphysema due to mucosal tears, unilateral soft tissue emphysema, and otitis media. Surgical emphysema is usually self-resolving, and otitis media is managed accordingly. However, the failure rate of BET is a major aftermath complication but is seen very minimally [31].

Even in the pediatric age range, pre-and postoperative satisfaction and ear-related complaints such as equalization of pressure, ear stress, hearing impairment, discomfort, and limitations in everyday living were examined. The majority of patients in research by Leichtle A et al. demonstrated improvements in their hearing impairment, auditory strain, restrictions on everyday activities, and contentment with repeated inflammations, which were supported by improved tubomanometry and tympanogram results [32]. When adenotomy, paracentesis, or grommets have failed in the past, BET in children has now proven to be a safe, effective, and promising way to treat persistent tube dysfunction [32]. Several studies have discovered that the improvement in ET function with BET does not happen right away but instead takes place over 6 to 8 weeks. This timeframe implies that a process of scarring or healing is taking place to enhance the function [33].

## Conclusions

For the treatment of eustachian tube dysfunction that has failed to respond to primary management, balloon dilation is a quick, easy, and secure procedure. The eustachian tube looks to be safely, affordably, and effectively dilated with balloons. The evidence from diverse data-gathering approaches, non-controlled case series, and short-term results are restricted to many recently developed procedures. Moreover, short-term evidence shows encouraging, favorable outcomes based on objective assessments, and balloon dilatation offers a vitally useful advancement when employed selectively in patients resistant to the most effective existing therapy. 
